# Beyond Microsystem Fixes: Targeting National Drivers of Low-Value Care: Comment on "Key Factors that Promote Low-Value Care: Views of Experts From the United States, Canada, and the Netherlands"

**DOI:** 10.34172/ijhpm.2022.7077

**Published:** 2022-08-06

**Authors:** Adina Weinerman, Christine Soong

**Affiliations:** ^1^Division of General Internal Medicine, Sunnybrook Health Sciences Centre, Toronto, ON, Canada.; ^2^Division of General Internal Medicine, Sinai Health, Toronto, ON, Canada.; ^3^Institute of Health Policy, Management and Evaluation, University of Toronto, Toronto, ON, Canada.; ^4^Centre for Quality Improvement and Patient Safety, University of Toronto, Toronto, ON, Canada.

**Keywords:** Low-Value Care, Overuse, Shared Decision-Making

## Abstract

Low-value care drivers and interventions are often focused on a microsystem (eg, clinic or inpatient ward) or within a health system. Identification of national drivers such as payment structure and medical culture of overuse can help identify regional approaches to reducing low-value care. However, these approaches in isolation are insufficient and require additional strategies. These can include policy and payment changes and adopting shared decision-making (SDM). SDM has the potential to move medical culture away from the ‘more is better’ paternalistic and physician-centric culture to one that actively engages patients as full partners in managing their care.

 Much of the research on low-value care focused on describing the magnitude of the problem with little in the way of effective, large-scale solutions. Many local, often single-centered context-specific solutions have been described, making it challenging to scale and spread. A regional or national approach to understanding drivers of low-value care and their associated solutions holds great appeal to quality improvement researchers and policy-makers.

 In the International Journal of Health Policy and Management, Verkerk and colleagues aimed to identify the key factors that impact low-value care on a national level. This was done by conducting semi-structured interviews with 18 experts on low-value care, from three countries (the United States, Canada, and the Netherlands) that are actively reducing low-value care.^1^ They described 7 distinct factors that promote low-value care across three different categories (system, knowledge and social). The system category included (1) payment structure, (2) pharmaceutical and medical device industry, and (3) fear of malpractice litigation. The knowledge category included (4) biased evidence of knowledge and (5) medical education. And the ‘more is better’ social category included (6) public culture and (7) medical culture. The authors concluded that interdependent factors regarding the healthcare system and culture lead to the provision of low-value care and that better awareness and understanding of these factors can help support policy changes that promote high-value care.

 The reason for focusing on national factors was based on prior evidence that reductions in low-value care is better achieved by changing systems and policies rather than trying to change clinician behaviour.^2,3^ However, several of the examples of promising strategies described to counter low-value care (eg, education, information campaigns and increasing awareness) do not target policy. On the contrary, these low-impact tools have a track-record of inefficacy.^4^ The other listed strategies of funding and reimbursement reform, compensating physicians for value rather than volume, are far more likely to be impactful and should be seriously considered by policy-makers.

 Included in the payment structure section, the authors suggest “moving away from pay for performance” in apparent reference to volume-based physician reimbursement. Interestingly, there was no exploration of pay-for-performance (“P4P” or “pay-for-results”) programs implemented across many countries, including those whose perspectives were highlighted in this study. The introduction of hospital P4Ps has generated numerous debates over its impact on clinical outcomes.^5,6^ Often criticized for incentivizing (or disincentivizing through penalties) narrow targets of quality such as process measures, P4Ps have at times spurred low-value care in their focus on timeliness measures at the expense of other domains of quality such as cost.^7,8^ For example, early P4Ps in the United States focusing on emergency department response times to early initiation of pneumonia treatment resulted in overuse of antimicrobials and inappropriate blood culture testing.^9^ Fortunately, recent evolution of reimbursement schemes toward incentivizing value appears to be a promising pivot towards achieving a more balanced quality agenda.^10^ As such, national healthcare payment and incentive programs remain a powerful influence on utilization and early P4P experiences provide an important lesson on the unintended consequence of achieving select quality targets at the expense of value.

 Another interesting proposed national driver of low-value care is the “more is better” public culture factor, which translates into patients and families requesting low-value care from their clinician. Some of the experts interviewed believe that this culture is a worldwide phenomenon and a significant factor promoting low-value care. This is highlighted in the following quote from the study: “the patient does not want to leave without a prescription with the idea that at least something has been done.” Although this may be true for a proportion of low-value care, there is no evidence to support that this is a *primary* national factor impacting low value care. As the authors acknowledge, there is a dominant, but over-emphasized *perception* that public culture is a significant factor. Inclusion of patients and carers in the study could have provided a more balanced perspective on perceived public culture.

 Rather than the public demanding more testing and treatment, it is more likely that healthcare workers underappreciate the harm associated with overtreatment and overestimate the benefits resulting in a failure to fully engaging the patient in an objective risk-benefit analysis. This may represent a form of paternalism in medical culture with a resultant transference of *perceived* desire for more care onto the patient. The clinician assumes that (*a*) the patient “wants something done”; and (*b*) the ‘something’ is a prescription for an antibiotic without a fulsome discussion on the appropriate management of a viral illness. In a survey about perceptions about Choosing Wisely recommendations that involved not performing a test or treatment for symptomatic patients (eg, antibiotics for sinusitis and imaging for low back pain), primary care providers anticipated major challenges in getting patients to accept these recommendations.^11^ This is in direct contradiction to studies demonstrating communication designed to shape patients’ mental models can have substantial effects on risk perception.^12^

 While it is true that the quality of information provided to the public likely overestimates benefits and underestimates harm and that society is less willing to accept risks or uncertainty, the literature is clear that in shared decision-making (SDM) utilizing patient-oriented material and decision aids, patient preferences are not drivers of low-value care on a national level.^13^ The authors focused on national rather than microsystem factors (such as lack of SDM) that promote low-value care. However, we would be remiss not to highlight SDM as one of the few patient engagement methods that have been shown to be effective in decreasing the use of low-value care.^14,15^ Decisions aids support patients by helping make their decisions more explicit and providing information about associated benefits/harms of available options. When decisions aids are used, they have been shown to increase the number of people choosing more conservative approaches (eg, conservative management over major elective invasive surgery and to avoid medically unnecessary screening tests) and improve patients’ knowledge and more accurate risk perceptions.^13^ Systematic reviews support the concept that when patients actually understand the available treatment options, they do not ask for more care with examples of reductions in antibiotic prescribing by 40% (compared to usual care) for acute respiratory infections in primary care without an increase in repeat consultations for the same illness or decreased satisfaction.^16^ Another meta-analysis demonstrated that patient-oriented education reduced the use of low-value care by an average of 31%.^14^

 An often-cited barrier to SDM is the perception that time constraints limit its utility and feasibility in many busy clinical settings. However, the evidence refutes this claim. In one study, the length of a consultation when decision aids were used increased by only 2.6 minutes compared to usual care.^13^ While we strongly support increasing the use of SDM, we acknowledge that for some patients requesting low-value care, it can be challenging to reassure them and that clinicians should have the skills to navigate these conversations in a time-efficient way. It is also helpful to think of strategies, eg, providing an antibiotic prescription is symptoms do not improve, or booking a follow-up appointment to ensure symptom resolution or providing factual information about “red flag symptoms.”

 The impact and importance of SDM has been enshrined in policy in the US where The Centers for Medicare & Medicaid Services made SDM a precondition for payment for a number of conditions and a number of states have passed legislature on decision-making for elective procedures.^17^ Other countries have yet to adopt similar policies although there is a call for nations to follow suit.^18^ So, in many ways, SDM may be considered a part of a national strategy to counteract the driver of a “more is better” paternalistic medical culture.

 In summary, Verkerk and colleagues have furthered our understanding of national drivers of low-value care. Policy-makers interested in national approaches to reduce low-value care should engage patients and carers to carefully craft quality policies and programs that incorporate value as a dimension of quality and consider incentivizing and enabling SDM by clinicians.

## Ethical issues

 Not applicable.

## Competing interests

 Authors declare that they have no competing interests.

## Authors’ contributions

 All authors participated in the development of the report, including conception, provision of references, writing of the manuscript, revision of the draft, and approval of the final version.
